# The neural correlates of apathy in the context of aging and brain disorders: a meta-analysis of neuroimaging studies

**DOI:** 10.3389/fnagi.2023.1181558

**Published:** 2023-06-16

**Authors:** Hongjie Yan, Huijun Wu, Zenglin Cai, Shouyun Du, Lejun Li, Bingchao Xu, Chunqi Chang, Nizhuan Wang

**Affiliations:** ^1^Department of Neurology, Affiliated Lianyungang Hospital of Xuzhou Medical University, Lianyungang, China; ^2^School of Biomedical Engineering, Health Science Center, Shenzhen University, Shenzhen, China; ^3^Department of Electrical and Electronic Engineering, The University of Hong Kong, Hong Kong, Hong Kong SAR, China; ^4^Department of Neurology, Suzhou Hospital, Affiliated Hospital of Medical School, Nanjing University, Suzhou, China; ^5^Department of Neurology, Gusu School, Suzhou Science and Technology Town Hospital, Nanjing Medical University, Suzhou, China; ^6^Department of Neurology, Guanyun People’s Hospital, Guanyun, China; ^7^Department of Neurology, Suzhou TCM Hospital Affiliated to Nanjing University of Chinese Medicine, Suzhou, China; ^8^Pengcheng Laboratory, Shenzhen, China; ^9^School of Biomedical Engineering, ShanghaiTech University, Shanghai, China

**Keywords:** apathy, meta-analysis, mental disorder, neurodegenerative disorder, traumatic brain injury, normal cognitive aging

## Abstract

**Introduction:**

Apathy is a prevalent mood disturbance that occurs in a wide range of populations, including those with normal cognitive aging, mental disorders, neurodegenerative disorders and traumatic brain injuries. Recently, neuroimaging technologies have been employed to elucidate the neural substrates underlying brain disorders accompanying apathy. However, the consistent neural correlates of apathy across normal aging and brain disorders are still unclear.

**Methods:**

This paper first provides a brief review of the neural mechanism of apathy in healthy elderly individuals, those with mental disorders, neurodegenerative disorders, and traumatic brain injuries. Further, following the preferred reporting items for systematic reviews and meta-analyses (PRISMA) guidelines, the structural and functional neuroimaging meta-analysis using activation likelihood estimation method is performed on the apathy group with brain disorders and the healthy elderly, aiming at exploring the neural correlates of apathy.

**Results:**

The structural neuroimaging meta-analysis showed that gray matter atrophy is associated with apathy in the bilateral precentral gyrus (BA 13/6), bilateral insula (BA 47), bilateral medial frontal gyrus (BA 11), bilateral inferior frontal gyrus, left caudate (putamen) and right anterior cingulate, while the functional neuroimaging meta-analysis suggested that the functional connectivity in putamen and lateral globus pallidus is correlated with apathy.

**Discussion:**

Through the neuroimaging meta-analysis, this study has identified the potential neural locations of apathy in terms of brain structure and function, which may offer valuable pathophysiological insights for developing more effective therapeutic interventions for affected patients.

## Highlights

- Structural gray matter atrophy in the precentral gyrus, bilateral insula, medial/inferior frontal gyrus, left caudate, and right anterior cingulate is associated with apathy;
- Functional connectivity in the putamen and lateral globus pallidus is correlated with apathy;
- The identified neural locations may serve as therapeutic targets for individuals with apathy.

## 1. Introduction

Apathy is commonly defined as a state of reduced feeling, emotion, and interest (Mann, 1990; Marin, 1991), where its association with the aging process (Fazio et al., 2016; Jang et al., 2021), various mental disorders (MD) (Alexopoulos et al., 2013; Yuen et al., 2014), neurodegenerative disorders (NDD) (Reijnders et al., 2010; Wei et al., 2020; Miller et al., 2021; Azocar et al., 2022), and traumatic brain injury (TBI) (Knutson et al., 2014; Hogeveen et al., 2021) suggests that it can serve as an independent risk factor for a range of diseases, including those affecting healthy elderly individuals (Eurelings et al., 2014). In this section, we first provide a concise review of the neural mechanisms underlying apathy, focusing on neuroimaging findings in the context of normal cognitive aging, mental disorder, neurodegenerative disorder, and traumatic brain injury. We also present a summary of key findings related to apathy across the healthy elderly and brain disorders in Table 1.

**TABLE 1:** A neuroimaging summary of the negative effects of apathy on normal cognitive aging, mental disorders, neurodegenerative disorders, and traumatic brain injury.

References	Diseases	*N*	Imaging technique	Apathy measure	Key findings
**Normal cognitive aging**					
Yan et al., 2015	Healthy elderly	36	sMRI	AS	Right putamen
Fazio et al., 2016	Healthy elderly	311	task-fMRI	AS	Dorsolateral prefrontal cortex
Jang et al., 2021	Healthy elderly	100	rs-fMRI	AS	Bilateral paracingulate gyrus; right insula; right temporal pole; anterior cingulate
**Mental disorders**					
Alexopoulos et al., 2013	Depression	26	rs-fMRI	AES	Amygdala; caudate; putamen; globus pallidus; thalamus; dorsolateral; ventrolateral prefrontal cortices
Yuen et al., 2014	Depression	16	rs-fMRI	AES	Right anterior insula; dorsal anterior cingulate
Lavretsky et al., 2007	Depression	43	sMRI	AES	Right anterior cingulate
Caravaggio et al., 2018	Schizophrenia	20	sMRI	SANS	Bilateral frontal inferior operculum; left precentral; left middle frontal gyrus
**Neurodegenerative disorders**					
Tunnard et al., 2011	AD	111	sMRI	NPI	Left caudal anterior cingulate cortex; left lateral orbitofrontal cortex; left superior/ventrolateral frontal regions
Kazui et al., 2017	AD	98	SPECT; sMRI	NPI	Right caudate nucleus; left posterior medial frontal lobe; right superior frontal lobe; bilateral culmen-fusiform gyri; left occipital lobe
Marshall et al., 2007	AD	41	PET	SANS	Bilateral anterior cingulate; medial orbitofrontal cortex; bilateral medial thalamus
Migneco et al., 2001	AD	41	SPECT	NPI	Bilateral anterior cingulate
Marshall et al., 2019	AD	40	PET	AES	Right anterior cingulate; dorsolateral prefrontal cortices
Bruen et al., 2008	AD	31	sMRI	NPI	Bilateral anterior cingulate; bilateral frontal cortex; bilateral putamen; left caudate
Kang et al., 2012	AD	18	SPECT	NPI	Right amygdala; right temporal gyrus; right posterior cingulate; right superior frontal gyrus; left inferior frontal gyrus; bilateral precentral
Stanton et al., 2013	AD	34	sMRI	IA	Ventromedial orbitofrontal cortex; left insula; anterior cingulate; ventrolateral orbitofrontal cortex
Wei et al., 2020	AD; bvFTD	92	sMRI	DAS	Bilateral frontal pole; left putamen; left orbitofrontal cortex; left insula; left inferior frontal gyrus
García-Alberca et al., 2019	AD	46	sMRI	NPI	Medial temporal lobe
Alzahrani et al., 2016	PD	65	sMRI	NPI	Left insula; left inferior/middle/medial frontal gyrus; left superior temporal gyrus; right anterior cingulate
Reijnders et al., 2010	PD	55	sMRI	AES; LARS	Bilateral precentral gyrus; bilateral inferior parietal/frontal gyrus; bilateral insula; bilateral posterior cingulate gyrus; right precuneus
Robert et al., 2006	PD	31	SPECT	AES	Right anterior cingulate
Baggio et al., 2015	PD	62	sMRI	AS	Left limbic frontal lobe; left precentral; left frontal pole; left paracingulate; left orbitofrontal cortex
Shin et al., 2017	PD	124	sMRI; FDG-PET	AS	Left precentral; left precuneus; left inferior parietal lobule
Shen et al., 2018	PD	42	rs-fMRI	AS	Bilateral superior frontal gyrus; left orbital middle frontal gyrus
Peters et al., 2006	fv-FTD	41	FDG-PET	NPI	Posterior orbitofrontal cortex
Kumfor et al., 2018	AD; bvFTD	150	sMRI	NPI	Bilateral insula; bilateral caudate; left putamen; left superior frontal gyrus; right medial frontal gyrus; right temporal pole
**Traumatic brain injury**					
Knutson et al., 2014	pTBIs	176	VLSM	NPI	Left insula; left middle frontal gyrus; left inferior frontal triangular; left superior frontal
Hogeveen et al., 2021	TBI	98	rs-fMRI	FrSBe	Right paracingulate gyrus

*N*, numbers; PD, Parkinson disease; AD, Alzheimer disease; TBI, traumatic brain injury; bvFTD, behavioral variant frontotemporal dementia; fv-FTD, frontal variant of frontotemporal dementia; pTBIs, penetrating traumatic brain injuries; VLSM, voxel-based lesion-symptom mapping; sMRI, structural magnetic resonance imaging; rs-fMRI, resting-state functional magnetic resonance imaging; task-fMRI, task functional magnetic resonance imaging; FDG-PET, fluoro-d-glucose positron emission tomography; SPECT, single-photon emission computerized tomography; AS, apathy scale; AES, apathy evaluation scale; LARS, lille apathy rating scale; NPI, neuropsychiatric inventory; SANS, scale for assessment of negative symptoms; FrSBe, frontal systems behavior scale; DAS, dimensional apathy scale; IA, apathy inventory.

As the global elderly population continues to increase rapidly, maintaining a “healthy” aging life has become an important concern. Unfortunately, apathy is a neuropsychiatric symptom with the prevalence rates ranging from 2 to 4.8% among cognitively normal older adults (Onyike et al., 2007; Geda et al., 2008; Lanctôt et al., 2017), which may affect their activities of daily living functioning and quality of life. Moreover, a 6 years follow-up study of 3,427 community-dwelling older people found that apathy was significantly associated with the risk of dementia, especially in individuals without cognitive impairment (van Dalen et al., 2018a). At the neural circuit level, research has revealed that apathy symptoms were associated with the gray matter volume in the prefrontal-basal-ganglia network (Yan et al., 2015), and the integrity of the frontal-subcortical network (Lanctôt et al., 2017). Studies have also demonstrated that healthy elderly individuals with apathy had smaller gray matter volumes in the frontal and temporal regions, parietal white matter volumes, thalamus volumes, and higher numbers of frontal white matter lesions (Grool et al., 2014). Therefore, age-related changes in the prefrontal cortex may make the elderly more vulnerable to apathy (Kawagoe et al., 2017).

Apathy is also a commonly observed symptom across various mental disorders (Steffens et al., 2022). For example, apathy is a negative symptom in schizophrenia, significantly reducing bilateral frontal lobe volumes happened in the high apathy group (Roth et al., 2004). Further, a replication neuroimaging study conducted by Burrer et al. (2020) showed that apathy is not only associated with reduced ventral striatal volume in schizophrenia, which also suggests that functional and structural striatal neuroimaging correlates of apathy can occur independently in schizophrenia (Burrer et al., 2020). Additionally, a negative correlation between the domain of apathy and the resting-state functional connectivity (rsFC) in the default mode network (DMN) has been observed (Forlim et al., 2020). Besides, it has been found that an increased level of apathy symptom mediates the relationship between cognition and depression (Funes et al., 2018). In depressed elderly patients with high levels of apathy, a decrease in rsFC of the nucleus accumbens (NAcc) with the amygdala, caudate, putamen, globus pallidus, and thalamus was observed, along with an increase in rsFC with the dorsomedial prefrontal cortex (dACC), superior frontal cortex, and insula compared to non-apathetic patients (Alexopoulos et al., 2013). Additionally, the apathetic subjects with late-life depression had lower saliency network rsFC and altered network FC patterns in right dorsolateral prefrontal cortex (DLPFC) nodes of the cognitive control network com-pared to older depressive patients without apathy (Yuen et al., 2014).

Moreover, there is growing neuroimaging evidence to suggest that apathy plays a significant role in various neurodegenerative disorders, such as Parkinson’s disease (PD), mild cognitive impairment (MCI), Alzheimer’s disease (AD), etc. Based on resting-state functional magnetic resonance imaging (rs-fMRI) technology, the study by Baggio et al. (2015) showed that the PD patients with apathy had decreased FC between the left striatal and frontal areas, and amongst PD patients’ apathy was inversely correlated with FC between the subdivisions of the left frontal lobe. Apart from PD, the MCI represents a transitional stage between healthy aging and dementia, with the prevalence of MCI estimated between 5.0 and 36.7% in the general older population (Sachdev et al., 2015; Ma, 2020), and apathy prevalence reported to range from 10.7 to 44.8% (Palmer et al., 2010; Richard et al., 2012). In a recent study conducted by Raimo et al. (2019), an activation likelihood estimation (ALE)-based meta-analysis (Eickhoff et al., 2009) was employed to investigate the neural underpinnings of apathy among individuals diagnosed with neurodegenerative disorders such as Fronto-Temporal Dementia (FTD), AD, and PD. The findings revealed a significant association between apathy and both hypometabolism and reduced gray matter volume specifically localized in the left inferior frontal gyrus (Raimo et al., 2019). A systematic review found that the apathy was associated with an approximately 2-fold increased risk of dementia in memory clinic patients (van Dalen et al., 2018b). Robert et al. (2006) and colleagues observed that MCI patients with apathy were more likely to develop into AD more than those without apathy. In the AD population, apathy has been linked to the reduced daily functioning, caregiver distress, and poor outcome. The study by Onyike et al. (2007) suggested that apathy is an early sign of cognitive decline. The emergence of an MCI plus apathy phenotype progresses to dementia (Bruen et al., 2008), and it is also possible that apathy precedes MCI, implying apathy as a potential target for treatment in AD (Mortby et al., 2022).

Besides, apathy is a prevalent symptom that occurs following TBI, which can cause severe cognitive impairment and negative psychosocial outcomes. Estimates of apathy following TBI range widely from 15 to 71% (Skidmore et al., 2015). It can be challenging to differentiate dysexecutive disorders from apathy after TBI because the cognitive aspects of apathy usually include executive functions associated with goal-directed behavior (Green et al., 2022). Alternatively, as the apathy is often characterized by a loss of interest in activities, some authors have suggested that deficits in sustained attention after frontal injury may be the main underlying factor (Daffner et al., 2000). TBI typically results in damage to orbitofrontal regions and often involves ventromedial areas of the prefrontal cortex, which can lead to decision-making deficits, as argued by Bechara (2004). In addition, different apathy profiles may arise from lateral prefrontal and medial prefrontal damage (Knutson et al., 2014).

The brief review presented above suggests that apathy may serve as an independent risk factor for a variety of brain diseases, as well as being observed in healthy elderly individuals. Building upon this literature, it is hypothesized that apathy may share a common neurophysiological mechanism among apathetic healthy elderly individuals and individuals affected by various brain diseases. In order to investigate the neurophysiological basis of apathy further, the present study next aims to conduct a meta-analysis utilizing neuroimaging studies related to apathy.

## 2. Meta-analyses of neuroimaging apathy

**FIGURE 1:**
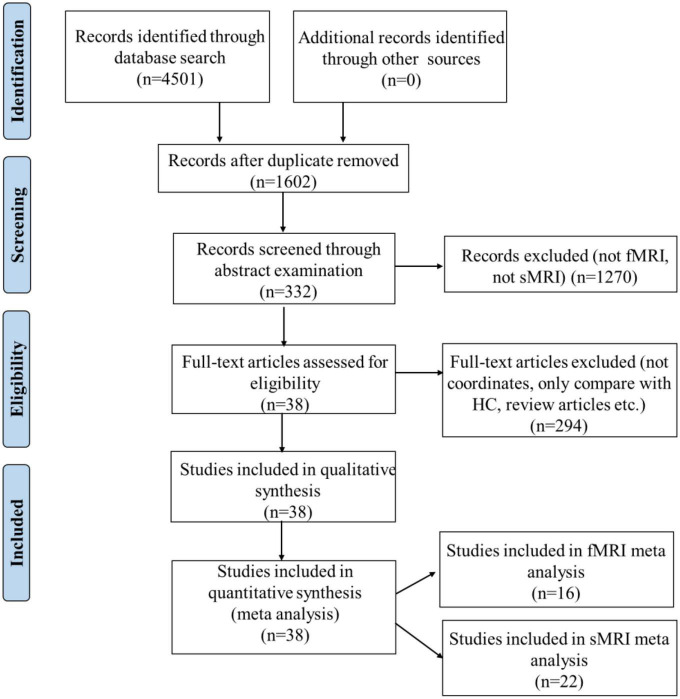
Preferred reporting items for systematic reviews and meta-analyses (PRISMA) flow diagram of study selection. HC, healthy controls.

A systematic selection of appropriate peer-reviewed studies was undertaken by searching the databases of PubMed, Google scholar, Web of Science and by checking references cited in each paper according to the standard preferred reporting items for systematic reviews and meta-analyses (PRISMA) procedure (Moher et al., 2009). The keyword combination is “apathy” AND “neuroimaging” OR “magnetic resonance imaging” OR “MRI” OR “functional magnetic resonance imaging” OR “fMRI” AND “Parkinson” OR “Alzheimer” OR “Dementia” OR “Huntington” OR “Mild Cognitive Impairment” OR “Mental disorder” OR “Psychiatric” OR “Depression” OR “Schizophrenia” OR “Traumatic Brain Injury” OR “Healthy Aged People.” The detailed procedure was presented in Figure 1.

### 2.1. ALE meta-analysis

Activation likelihood estimation is a widely-used meta-analytic technique in neuroimaging to combine multiple studies, and to identify brain regions that consistently show activation across experiments (Turkeltaub et al., 2002; Laird et al., 2005, 2009a,b). Briefly, the ALE procedure involves the following steps:

1. Data collection: Gather a set of studies that investigate a specific condition using neuroimaging techniques such as magnetic resonance imaging (MRI), functional MRI (fMRI) or positron emission tomography (PET).
2. Coordinate extraction: Identify the peak coordinates of activation reported in each study. These coordinates represent the locations of maximum brain activity associated with the task or condition of interest.
3. Modeling activation: Create three-dimensional Gaussian probability distributions centered on each peak coordinate. The size of these distributions represents the spatial uncertainty associated with the reported activations.
4. Spatial modeling: Combine the individual activation models to create an overall activation likelihood map. This map represents the likelihood of activation at each voxel in the brain across all studies.
5. Activation likelihood estimation calculation: Calculate the ALE score for each voxel by determining the proportion of overlapping activation likelihoods from different studies.
6. Thresholding: Establish a significance threshold to determine which voxels show activation beyond what would be expected by chance. Various statistical methods can be used to determine the threshold, such as permutation testing, false discovery rate correction, etc.
7. Cluster analysis: Identify clusters of significant activation by grouping adjacent activated voxels. This step helps to reduce the likelihood of false-positive findings.
8. Interpretation: Analyze the clusters of significant activation to infer brain regions that consistently exhibit activation across the included studies.

Studies were included in this meta-analysis compared subjects with or without apathy (e.g., healthy elderly with apathy vs. healthy elderly without apathy, AD with apathy vs. AD without apathy, etc.). Studies focused on functional connectivity/gray matter volume alterations associated with apathy. The criterion of apathy diagnosis was clearly reported in the involved studies (listed in Tables 2, 3) in this meta-analysis, which was subject to different apathy evaluation questionnaires such as Apathy Scale (AS) (Starkstein et al., 1992), Apathy Evaluation Scale (AES) (Marin et al., 1991), Neuropsychiatric Inventory (NPI) (Cummings, 2020), etc. We performed an ALE analysis using the GingerALE software (Version 3.0.2) (Eickhoff et al., 2009, 2011, 2012; Turkeltaub et al., 2012). The resulting *p*-values were threshold at *p* = 0.05 with 1,000 threshold permutations, cluster-level family-wise error (cFWE) with cluster-forming threshold at *p* < 0.001. Based on a recent simulation study (Eickhoff et al., 2016), a recommendation was made to include at least 17–20 experiments in ALE meta-analyses in order to have sufficient power to detect smaller effects and to also make sure that results are not driven by single experiments. The ALE mapping results were displayed on the MNI template using Mircron^1^.

**TABLE 2:** Original structural magnetic resonance imaging (MRI) studies included in the structural neuroimaging meta-analysis.

References	Population	*N* (F)	Apathy cases (F)	Age (SD)	Imaging technique	Analysis	Apathy measure	Foci
Alzahrani et al., 2016	PD	89 (52)	25	68.7 (8.4)	sMRI	VBM; WB	NPI	9
Apostolova et al., 2007	AD	35 (20)	17(10)	73.9 (2.25)	sMRI	VBM; WB	NPI	3
Bertoux et al., 2012	bvFTD	20 (10)	–	68.5 (9.1)	sMRI	VBM; ROI	SEA	1
Bruen et al., 2008	AD	31 (12)	31 (12)	77.1 (8.6)	sMRI	VBM; WB	NPI	11
Caga et al., 2021	ALS	109 (39)	30	63.1 (1.5)	sMRI	VBM; WB	DAS	7
Caravaggio et al., 2018	Schizophrenia	20 (0)	20 (0)	42.75 (11.82)	sMRI	VBM; WB	SANS	15
De Paepe et al., 2021	HD	45 (31)	45 (31)	45.56 (11.9)	sMRI	VBM; ROI	PBA-s	5
Eslinger et al., 2012	FTD	42	26	65.3(12.6)	sMRI	VBM; WB	AES	4
Kumfor et al., 2018	AD; bvFTD	150 (73)	90	62.9 (7.9)	sMRI	WB	NPI	25
Martinez-Horta et al., 2017	PD	36 (20)	18 (10)	68.8 (10.1)	sMRI	VBM; WB	UPDRS	7
Massimo et al., 2009	FTLD	67 (29)	9 (2)	63 (10.6)	sMRI	WB	NPI	10
Nour et al., 2021	AD	105 (50)	24 (8)	77.5 (6.8)	sMRI	VBM; WB	NPI-Q	7
Powers et al., 2014	bvFTD	45	11	60.5 (2)	sMRI; DWI	VBM; WB	NPI	2
Prange et al., 2019	PD	39 (14)	14 (3)	62.5 (10.2)	sMRI; DTI	VBM; WB	LARS; AS	3
Reijnders et al., 2010	PD	55	55	62 (10.1)	sMRI	VBM; WB	LARS; AES; NPI	28
Rosen et al., 2005	FTD; PSP	148	–	64.8 (9.4)	sMRI	VBM; ROI	NPI	1
Shin et al., 2017	PD	31 (13)	12 (6)	73.8 (3.4)	sMRI; FDG-PET	VBM; WB	AS	4
Stanton et al., 2013	AD; PSP	34 (16)	17 (7)	73.9 (6.5)	sMRI	VBM; WB	AES; NPI	4
Terada et al., 2018	PD	110 (59)	40 (24)	64.7 (8)	sMRI	VBM; WB	FrSBe	1
Wei et al., 2020	AD; bvFTD	92 (32)	73	67.44 (9.5)	sMRI	VBM; WB	DAS	21
Yan et al., 2015	Healthy elderly	36 (17)	19 (8)	63.7 (3)	sMRI	VBM; WB	AS	2
Zamboni et al., 2008	FTD	76 (40)	62 (33)	61.2 (1)	sMRI	VBM; WB	FrSBe	12

*N*, numbers; F, females; SD, standard deviation; PD, Parkinson disease; AD, Alzheimer disease; bvFTD, behavioral variant frontotemporal dementia; FTD, frontotemporal dementia; PSP, progressive supranuclear palsy; FTLD, frontotemporal lobar degeneration; ALS, amyotrophic lateral sclerosis; HD, Huntington’s disease; sMRI, structural magnetic resonance imaging; FDG-PET, fluoro-d-glucose positron emission tomography; DWI, diffusion weighted imaging; DTI, diffusion tensor imaging; VBM, voxel-based morphometry; AS, apathy scale; AES, apathy evaluation scale; LARS, lille apathy rating scale; NPI, neuropsychiatric inventory; SANS, scale for assessment of negative symptoms; FrSBe, frontal systems behavior scale; DAS, dimensional apathy scale; NPI-Q, neuropsychiatric inventory questionnaire; UPDRS, unified Parkinson’s disease rating scale; PBA-s, short-problem behavior assessment; SEA, social cognition and emotional assessment.

**TABLE 3:** Original functional magnetic resonance imaging (MRI) studies included in the functional neuroimaging meta-analysis.

References	Population	*N* (F)	Apathy cases (F)	Age (SD)	Imaging technique	Analysis	Apathy measure	Foci
Alexopoulos et al., 2013	Depression	26	7	69.9 (4.9)	rs-fMRI	rsFC; ROI	AES	17
Baggio et al., 2015	PD	62 (25)	25 (5)	65.6 (12.89)	rs-fMRI	rsFC; ROI	AS	11
Büyükgök et al., 2020	AD	20 (12)	10 (6)	73	rs-fMRI	rsFC; ROI	AES	3
Fazio et al., 2016	Healthy elderly	311 (165)	–	27.3 (6.79)	task-fMRI	PPI/FC; ROI	AS	2
Forlim et al., 2020	Schizophrenia	76 (31)	35 (14)	35.3 (10.8)	rs-fMRI	ICA; ROI	SANS	1
Hamada et al., 2021	Healthy elderly	36 (16)	18 (7)	63.7 (3)	rs-fMRI	rsFC; ROI	AS	1
Hogeveen et al., 2021	TBI	98 (39)	70 (25)	32.6 (12)	rs-fMRI	rsFC; ROI	FrSBe	1
Jang et al., 2021	Healthy elderly	48 (18)	–	70.9 (7.88)	rs-fMRI	rsFC; ROI	AES	2
Joo et al., 2017	aMCI	100 (55)	50 (27)	72.1 (3.8)	rs-fMRI	rsFC; ROI	IA	1
Kawagoe et al., 2017	Healthy elderly	100 (56)	25 (18)	72.6 (3.9)	rs-fMRI	rsFC; ROI	AS	1
Shen et al., 2018	PD	61 (20)	20 (4)	63.35 (8.52)	rs-fMRI	ALFF; WB	AS	3
Skidmore et al., 2013	PD	15 (3)	–	62 (9)	rs-fMRI	ALFF; WB	LARS	5
Sun et al., 2020b	PD	46 (18)	20 (6)	59.85 (8.92)	rs-fMRI	Reho; WB	AS	2
Sun et al., 2020a	PD	46(18)	20(6)	59.85(8.92)	rs-fMRI	ALFF; WB	AS	8
Yuen et al., 2014	Depression	26	7	69.9 (4.9)	rs-fMRI	rsFC; ROI	AES	10
Zhao et al., 2014	AD	13	7	72.3 (7.55)	task-fMRI	Facial Expression; ROI	NPI; AES–C; LARS	4

*N*, numbers, F, females; PD, Parkinson disease; AD, Alzheimer disease; aMCI, amnestic mild cognitive impairment; TBI, traumatic brain injury; AS, apathy scale; AES, apathy evaluation scale; AES–C, apathy evaluation scale-clinical version; LARS, lille apathy rating scale; NPI, neuropsychiatric inventory; IA, apathy inventory; SANS, scale for assessment of negative symptoms; FrSBe, frontal systems behavior scale; FC, functional connectivity; Reho, regional homogeneity; ALFF, amplitude of low frequency fluctuations; ICA, independent component analysis; PPI, psychophysiological interactions; WB, whole brain; ROI, regions of interest.

According to the PRISMA flow diagram, there are 22 studies included 31 experiments from 1,578 patients and reported 182 foci of gray matter volume decreases associated with apathy (see Table 2).

According to the PRISMA flow diagram, there are 16 studies included 20 experiments from 706 patients and reported 73 foci of functional connectivity/ALFF decreases associated with apathy (see Table 3).

## 3. Results

**FIGURE 2:**
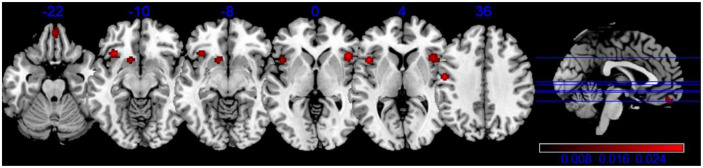
Activation activation likelihood estimation (ALE) map for gray matter atrophy associated with apathy. The ALE map significance was tested by using 1,000 permutations with a cluster-forming threshold of *p* < 0.001, and was corrected with a cluster-level family-wise error threshold of *p* < 0.05.

Our meta-analysis of structural MRI studies revealed six cluster of significant convergence between the studies, and they were in left Precentral Gyrus (BA 13/6), right Insula (BA 47), right Medial Frontal Gyrus (BA 11), left Inferior Frontal Gyrus and left Caudate (Putamen), which are shown in Table 4, Figure 2.

**TABLE 4:** Strengthened activation results in the apathy-free group in contrast to the apathy group in structural neuroimaging meta-analysis.

Volume (mm^3^)	MNI			Label	Brodmann area
	**x**	**y**	**z**		
1,656	−44	14	4	L insula/precentral gyrus/middle frontal gyrus	BA 13/44
1,640	44	20	0	R insula/inferior frontal gyrus/precentral gyrus	BA 13/47
912	2	50	−22	R medial frontal gyrus/anterior cingulate	BA 11/10
816	−34	22	−10	L inferior frontal gyrus/insula	BA 47
712	−12	14	−8	L caudate	Caudate Head/Putamen
656	−52	−8	36	L precentral gyrus/postcentral gyrus	BA 6/4

R, right hemisphere; L, left hemisphere; BA, brodmann areas; MNI, Montreal Neurological Institute.

**FIGURE 3:**
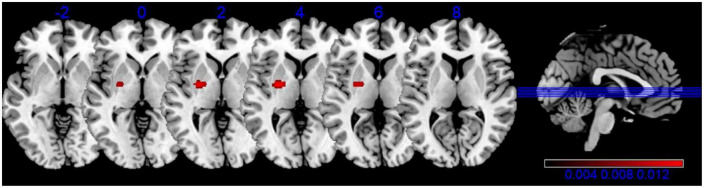
Activation activation likelihood estimation (ALE) map for the decreased brain functional connectivity associated with apathy. The ALE map significance was tested by using 1,000 permutations with a cluster-forming threshold of *p* < 0.001, and was corrected with a cluster-level family-wise error threshold of *p* < 0.05.

Our analysis of functional neuroimaging techniques revealed that Lentiform (Putamen) was correlated with apathy. The results indicated one significant cluster in this contrast, and they were located in Putamen and Lateral Globus Pallidus, shown in Table 5, Figure 3.

**TABLE 5:** Strengthened activation results in the apathy-free group in contrast to the apathy group in functional neuroimaging meta-analysis.

Volume (mm^3^)	MNI			Label	Brodmann area
	**x**	**y**	**z**		
704	−26	6	4	L lentiform nucleus	Putamen
	−20	−6	4	L lentiform nucleus	Lateral globus pallidus

R, right hemisphere; L, left hemisphere; BA, brodmann areas; MNI, Montreal Neurological Institute.

## 4. Discussion

Apathy is a prevalent mood disturbance, being widely distributed among the normal aging, mental disorders, neurodegenerative disorders and traumatic brain injuries. The aim of our study was to reveal the neural basis of apathy between the non-apathy and apathy groups across diverse populations, including healthy elderly, mental disorders, neurodegenerative disorders, acquired brain injuries. Despite the differences among these populations, the manifestations of apathy symptoms are generally consistent, and apathy is widely recognized as a common and incapacitating condition in various disorders, including AD, PD, FTD, TBI and so on Kos et al. (2016).

### 4.1. Altered structural and functional correlation of apathy

In our study, the apathy group exhibited structural atrophy in brain regions including the bilateral insula, precentral gyrus, inferior frontal gyrus, medial frontal gyrus, left caudate, postcentral gyrus, and right anterior cingulate, compared to the non-apathy group. Furthermore, we observed inverse correlations between apathy and brain functional connectivity, specifically in regions such as the left putamen and lateral globus pallidus. These findings suggest significant atrophy and dysfunction in the intrinsic neural activity of these regions in apathetic patients.

A recent ALE meta-analysis investigated the neural correlates of apathy in patients with neurodegenerative disorders, revealing a link between apathy and hypometabolism as well as reduced gray matter volume in the left inferior frontal gyrus (Raimo et al., 2019). Our study expands upon this research in several notable ways. Firstly, we included a larger subgroup of subjects with apathy, encompassing healthy elderly individuals, those with acquired brain injury, mental disorders, in addition to neurodegenerative disorder patients. Secondly, we employed stricter selection criteria by focusing exclusively on MRI/fMRI-based studies for the meta-analysis. Lastly, our study identified additional brain regions associated with apathy, extending beyond the left inferior frontal gyrus, through structural neuroimaging meta-analysis.

The putamen, particularly the caudate head, plays a role in the executive function of the fronto-striatal network (Baggio et al., 2015; Lucas-Jiménez et al., 2018). A SPECT study found dopaminergic neuronal loss in the bilateral putamen of patients with apathy, including those with Alzheimer’s disease and dementia with Lewy bodies (David et al., 2008). Additionally, the anterior cingulate circuit, part of the frontal-striatal circuits described in the model by Tekin and Cummings (2002), consists of a feed-forward loop from frontal cortical areas to the caudate nucleus and putamen (Mesulam, 2000). Another study observed abnormalities in the left putamen/ventral striatum following negative feedback (Waltz et al., 2013). Together, these findings partially support our results and suggest that apathy may arise from aberrant processing of reward stimuli and anticipation.

Our findings also revealed atrophy in the bilateral insula, a frontal lobe region involved in inhibitory control, body representation, and subjective emotional experience (Damasio, 1996). The observed insular damage in our study aligns with previous findings on apathy in neurodegenerative disorders and normal aging (Yuen et al., 2014; Kumfor et al., 2018; Jang et al., 2021). For instance, Moon et al. (2014) identified a negative correlation between apathy and volume of the bilateral anterior insular cortex. Moreover, a task-based fMRI study investigating cognitive and emotional empathy identified the precentral gyrus (BA 4) and right insula (BA 13) as potentially important components of emotional empathy (Kim et al., 2020). Additionally, mutism has been associated with right insula damage in stroke patients (Berthier et al., 1987; Gasquoine, 2014).

Significantly decreased volumes were observed in the bilateral precentral gyrus (more prominently in the left hemisphere), inferior frontal gyrus, and medial frontal gyrus in our study. According to Frijda (2010), emotions play a role in both the generation and execution/control of actions. Furthermore, Reijnders et al. (2010) found a correlation between high apathy scores and decreased gray matter density in the bilateral precentral gyrus. These brain imaging findings suggest the involvement of the precentral gyrus in both emotional and cognitive processes. Raimo et al. (2019) investigated structural and metabolic alterations associated with apathy across AD, FTD, and PD, and demonstrated that apathy was mainly associated with the left inferior frontal gyrus, which is strongly connected to a frontal-subcortical circuit involved in action planning and purpose generation (Levy and Dubois, 2006). The medial frontal gyrus (BA 10) is believed to play a crucial role in linking affect or emotional information with planned or ongoing behaviors (Kringelbach, 2005). The right medial frontal gyrus (BA 11/10) may also have a fundamental role in the development of apathy due to its functional and structural connectivity (Moayedi et al., 2015; Ray et al., 2015). Hence, abnormal changes in these brain areas may be associated with apathy symptoms.

### 4.2. Target areas for apathy-related neuroplasticity modulation

The structural and functional changes associated with apathy may indicate crucial brain regions for the treatment of apathy-related brain disorders. Our research robustly identified certain brain regions (refer to Figures 2, 3) through meta-analysis that consistently exhibited apathy-related alterations across both healthy elderly individuals and those with brain disorders. These commonly identified regions, displaying structural or functional changes, could be targeted for treatment of apathy-related brain disorders using neuromodulation techniques such as transcranial magnetic stimulation (TMS) (Kobayashi and Pascual-Leone, 2003; Hallett, 2007) and transcranial direct current stimulation (tDCS) (Paulus, 2011). For instance, Reis et al. (2008) conducted a comprehensive review of relevant studies and observed that these stimulation techniques could regulate memory formation and motor learning in healthy individuals. Furthermore, several studies have demonstrated the effectiveness of these stimulation techniques in treating mental disorders such as major depressive disorder and schizophrenia (Reis et al., 2008; Liu et al., 2017; Kennedy et al., 2018; Osoegawa et al., 2018), traumatic brain injury (Dhaliwal et al., 2015; Zhang et al., 2020), and neurodegenerative disorders (Elder and Taylor, 2014; Di Lorenzo et al., 2022). Specifically, Levkovitz et al. (2011) found that deep TMS applied over the prefrontal cortex led to remission of apathy in one-third of depressive patients with moderate apathy. Conversely, Suemoto et al. (2014) found no effect on apathy in elderly patients with moderate Alzheimer’s disease following repeated anodal tDCS over the left dorsolateral prefrontal cortex. Hence, based on previous studies, the brain regions identified (listed in Tables 4 and 5) are likely to be targeted for neural modulation of apathy-related neuroplasticity at the individual or group level in future.

### 4.3. Other neuroimaging studies of apathy

Other neuroimaging techniques used in studying apathy among the aging population include positron emission tomography (PET) (Marshall et al., 2007), single-photon emission computerized tomography (SPECT) (Benoit et al., 2004; David et al., 2008), and diffusion tensor imaging (DTI) (Prange et al., 2019). Early SPECT studies have shown a correlation between apathy and reduced regional cerebral blood flow in the orbitofrontal cortex (DeKosky and Scheff, 1990) and anterior cingulate (Migneco et al., 2001). Additionally, PET amyloid imaging using (11C) PiB has indicated a link between apathy and the accumulation of fibrillar amyloid in specific subregions of the prefrontal cortex (PFC), including the orbital, ventromedial, and polar PFC, as well as the anterior cingulate (Mori et al., 2014). Moreover, DTI analysis has demonstrated lower fractional anisotropy values in the left anterior cingulate of the apathy group compared to the non-apathy group (Kim et al., 2011; Tighe et al., 2012).

### 4.4. Limitations and future research

This study focuses exclusively on identifying the shared neural mechanism underlying apathy in normal aging and brain disorders, providing potential neural correlates for apathy. However, further investigations are necessary to address the following aspects: (1) Understanding the interaction between functional and structural changes is essential, as our analysis revealed apathy-related alterations in separate domains. (2) Exploring the evolving mechanism and role of apathy in the aging process and progression of brain disorders is crucial. (3) Although our ALE meta-analysis employed strict criteria for literature selection, the reliability of the findings may still be influenced by factors such as sample size, populations, comorbidities, medications, and number of studies. Therefore, careful validation of these apathy-related findings is required in future research. (4) Future studies should consider exploring the neural correlates of apathy specific to individual brain disorders through meta-analysis, once a sufficient number of related studies have been accumulated.

## 5. Conclusion

In summary, our meta-analysis study indicates that the neural correlates of apathy exist across normal aging and various brain disorders, suggesting that it may serve as an independent risk factor for brain disorders. Specifically, we noted that the putamen area was significantly activated in both the structural and functional meta-analysis, indicating a close correlation with apathy. Additionally, our structural meta-analysis revealed that gray matter atrophy in the Precentral Gyrus, Insula, Medial Frontal Gyrus, and Inferior Frontal Gyrus was also associated with apathy. These observed changes in structural/functional activation associated with apathy provide promising pathophysiological insights that have the potential to guide the development of more efficacious therapeutic interventions for brain disorders.

## Author contributions

HY, HW, and ZC: conceptualization, methodology, validation, formal analysis, and writing–original draft. ZC and HY: funding acquisition. SD and LL: investigation, idea discussion, and writing–review and editing. BX, CC, and NW: conceptualization, resources, writing–review and editing, supervision, funding acquisition, and project administration. All authors contributed to the article and approved the submitted version.

## Footnotes

1
https://www.nitrc.org/projects/mricron
